# Associations Between Intrinsic Capacity Profiles and Unmet Home- and Community-Based Care Need Patterns Among Older Adults in China: A Cross-Sectional Study

**DOI:** 10.3390/healthcare14203412

**Published:** 2026-10-13

**Authors:** Siyi Qin, Yang Luo, Miao Lin, Zhuonan Wu, Hui Chen, Guorong Wang

**Affiliations:** 1 College of Nursing, Chengdu University of Traditional Chinese Medicine, Chengdu 611137, China; 2 Nursing Department, West China School of Public Health, West China Fourth Hospital, Sichuan University, Chengdu 610041, China

**Keywords:** intrinsic capacity, unmet needs, home- and community-based care, older adults, latent class analysis, China

## Abstract

**Background:** Home- and community-based care services are expanding in China, but older adults differ substantially in both functional capacity and unmet service needs. This study aimed to identify intrinsic-capacity profiles and patterns of unmet home- and community-based care needs and to examine their associations among community-dwelling older adults in China. **Methods:** Data were obtained from 10,405 adults aged ≥65 years in the 2018 Chinese Longitudinal Healthy Longevity Survey. Intrinsic-capacity profiles were identified using scale-aware finite-mixture modeling with continuous and ordinal indicators, and unmet-need patterns were identified using latent class analysis. Associations between the two classifications were examined using chi-square tests and multinomial logistic regression. Missing covariate data were handled using 20 multiple imputations, and regression estimates were pooled using Rubin’s rules. **Results:** Five intrinsic-capacity profiles and three unmet-need patterns were identified. The overall crude association between intrinsic-capacity profiles and unmet-need patterns was very small and not statistically significant (χ^2^(8) = 14.77, *p* = 0.064; Cramér’s V = 0.027). In the primary adjusted model, the Cognition-Preserved Locomotion–Psychological Vulnerability Profile showed the most consistent association with Pattern 1 versus Pattern 3 (OR = 1.30, 95% CI 1.13–1.49), whereas the Very Severe Multidomain Impairment Profile showed a weaker nominal association (OR = 1.40, 95% CI 1.02–1.92) that was not maintained in the extended model. Given the multiple profile contrasts examined, these coefficient-level findings should be interpreted cautiously. No intrinsic-capacity profile was significantly associated with the Low-Need/Light Health Protection Pattern versus the High-Need pattern. **Conclusions:** Both intrinsic capacity and unmet home- and community-based care needs showed substantial heterogeneity. Intrinsic-capacity profiles alone did not correspond closely to unmet-need patterns, although a specific adjusted association was observed. Integrating intrinsic-capacity assessment with direct evaluation of service needs and broader social and health-related factors may provide a more appropriate basis for person-centered community care planning.

## 1. Introduction

Population aging is accelerating worldwide, placing substantial pressure on health and social care systems [1]. China, which has the largest older population globally, is moving rapidly from a moderately aged to a severely aged society [2]. By the end of 2024, the population aged 65 years and older in China had reached 220 million, accounting for 15.6% of the total population; the old-age dependency ratio was 22.8% [3]. By 2050, China’s older population is projected to exceed 500 million, making it one of the most aged societies in the world [4]. At the same time, socioeconomic development and changing family structures have weakened the traditional family-based model of eldercare, increasing the demand for professional and community-based care services [5]. Meeting these growing care needs is essential for improving the well-being of older adults and advancing healthy aging.

Home- and community-based care is an important strategy for supporting aging in place because it integrates resources from families, communities, and government systems. Since the 12th Five-Year Plan established the framework of home-based care as the foundation, community-based care as the support, and institutional care as the supplement, China has continued to expand services for older adults. Nevertheless, low utilization, regional inequalities, and structural mismatches between supply and demand remain prominent [6,7]. A national survey showed that only about one-third of older adults were satisfied with community services, whereas more than half had never used such services or reported that relevant services were unavailable in their communities [2]. This mismatch not only reduces the efficiency of resource allocation but may also adversely affect the physical and psychological health and quality of life of older adults [8,9]. Therefore, identifying differentiated service needs and providing integrated, targeted services are crucial for improving the effectiveness of home- and community-based care.

The World Health Organization (WHO) defines healthy aging as the process of developing and maintaining functional ability in later life [10]. A central component of this framework is intrinsic capacity, which refers to the composite of an individual’s physical and mental capacities across five domains: cognition, locomotion, vitality, psychological capacity, and sensory capacity [11]. Intrinsic capacity interacts with the environment to determine actual functioning [11]. Evidence suggests that intrinsic capacity is important for maintaining independent living, predicting disability, and shaping health outcomes [12,13]. Declines in intrinsic capacity may increase the need for daily assistance and medical care [14,15], and different capacity profiles may be associated with different patterns of service demand [16]. However, when community services fail to respond to functional decline in a timely and appropriate manner, older adults may experience unmet needs. If such needs persist, they may accelerate functional decline and increase the risk of disability [17,18]. Identifying unmet needs among older adults with different intrinsic capacity profiles is therefore essential for proactive needs assessment and targeted service planning. This approach is consistent with global healthy aging strategies [11,19] and with the WHO concept of capacity-based care, which emphasizes prevention and capacity enhancement for those with higher capacity, maintenance and delay of decline for those with emerging impairment, and dignity-oriented support and disease management for those with severe impairment [10]. On this basis, health and care systems can use comprehensive assessments to develop individualized support plans and promote social participation, quality of life, and healthy aging [10,20].

Previous studies have generally examined intrinsic capacity and care needs separately or have focused on individual service needs rather than multidimensional need patterns. Consequently, it remains unclear whether distinct patterns of intrinsic capacity correspond to distinct configurations of unmet home- and community-based care needs. Jointly identifying heterogeneity in both constructs may provide a more person-centered understanding of how functional capacity and perceived service gaps coexist in later life. Using data from the 2018 Chinese Longitudinal Healthy Longevity Survey (CLHLS), this study aimed to examine the heterogeneity of intrinsic capacity and unmet home- and community-based care needs among community-dwelling older adults in China and to explore the associations between these two multidimensional constructs. Specifically, we sought to: (1) identify distinct intrinsic-capacity profiles; (2) identify latent patterns of unmet home- and community-based care needs; and (3) examine whether intrinsic-capacity profiles were associated with unmet-need patterns after accounting for relevant sociodemographic, socioeconomic, social-security, intergenerational-support, and health-related factors. By jointly examining capacity profiles and unmet-need patterns, this study aimed to provide empirical evidence for a more differentiated understanding of service needs among older adults and to inform person-centered needs assessment and community-care planning.

## 2. Methods

### 2.1. Study Design and Population

The Chinese Longitudinal Healthy Longevity Survey (CLHLS) was launched in 1998 as a nationwide longitudinal survey of older adults in China. The 2018 survey covered more than 500 sample sites across 22 provinces, municipalities, and autonomous regions and included adults aged 65 years and older. Details of the sampling procedures and data quality have been published elsewhere [21]. The CLHLS was approved by the institutional review boards of Peking University and Duke University (IRB00001052-13074), and written informed consent was obtained from all participants or their legal representatives. This cross-sectional study used data from the 2018 wave of the CLHLS.

**Figure 1:**
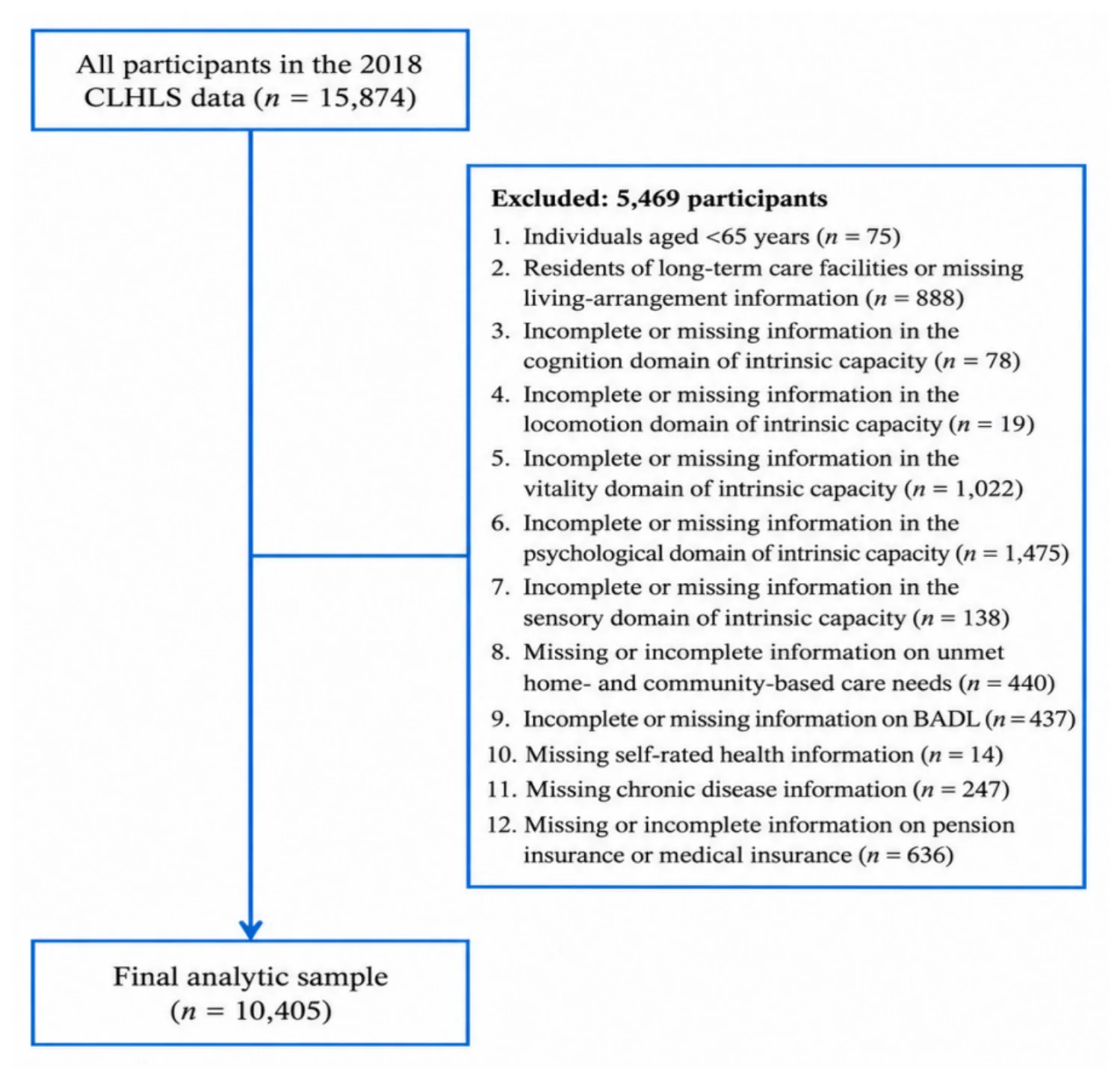
Flow diagram of participant selection.

Participants aged 65 years and older were eligible for inclusion. Participants were subsequently excluded in a sequential manner for institutional residence or missing living-arrangement information; insufficient data for the cognition, locomotion, vitality, psychological, or sensory domains of intrinsic capacity; incomplete information on unmet home- and community-based care needs; missing BADL scores; missing self-rated health information; missing chronic-disease information; missing pension information; or missing medical-insurance information. Detailed sequential exclusions are shown in Figure 1. After 5469 participants were excluded during this process, 10,405 community-dwelling older adults were included in the final analytic sample. Characteristics of included and excluded participants were additionally compared to evaluate potential selection bias (Supplementary Table S1).

### 2.2. Measurement

#### 2.2.1. Intrinsic Capacity

At the end of 2017, the World Health Organization released the Integrated Care for Older People (ICOPE) framework to support the systematic screening, assessment, and management of declines in intrinsic capacity among older adults [22]. Following the ICOPE framework and relevant prior studies, this study assessed intrinsic capacity across five domains: cognition, locomotion, vitality, psychological capacity, and sensory function [23].

Cognition: Cognitive function was assessed using the CLHLS cognitive battery adapted from the Mini-Mental State Examination (MMSE). The assessment covered seven components: orientation, immediate registration, attention and calculation, delayed recall, language and command following, visual construction, and verbal fluency/food naming. Most component items in the CLHLS questionnaire distinguish correct responses, incorrect responses, and questionnaire-coded inability to answer or perform the task. Consistent with established scoring practice in previous CLHLS studies, correct responses to the 23 dichotomously scored items contributed one point, whereas incorrect responses and responses coded as “unable to answer” or “unable to perform” did not contribute a point; the food-naming task contributed up to seven points. The resulting total score ranged from 0 to 30, with higher scores indicating better cognitive function [24,25].

To avoid deriving a cognitive-domain score from substantially incomplete assessments, a prespecified minimum-completeness criterion was applied. Participants with fewer than 18 of the 24 component variables containing a recorded response were treated as having missing cognitive data. Thus, the scoring procedure distinguished questionnaire-coded inability to answer or perform from true item-level missingness and did not involve blanket replacement of all missing MMSE data with zero. Because cognition was modeled as a continuous indicator in the present mixture analysis, no dichotomous MMSE cut-off was used to define cognitive impairment.

Locomotion: In this secondary-data analysis, locomotion was operationalized using three functional items available in the CLHLS that captured chair-rise ability, walking endurance, and repeated lower-extremity movement: (1) the ability to rise independently from a chair; (2) the ability to walk continuously for 2 li (approximately 1 km); and (3) the ability to complete three consecutive crouch-to-stand movements. For the chair-rise item, participants were classified as able to rise without assistance, able to rise with assistance or support, or unable to rise. For the walking and crouch-to-stand items, responses were classified as able independently, able with some difficulty or assistance, or unable. The three indicators were scored 2, 1, and 0, respectively, yielding a total locomotion score ranging from 0 to 6, with higher scores indicating better locomotor capacity.

A prespecified completeness rule was applied to limited item-level missingness. When two or more of the three component items were missing, the locomotion-domain score was treated as missing. When only one item was missing, the unobserved item was assigned the lowest score rather than estimating the unobserved performance from the remaining two items. This rule was applied uniformly and affected 79 participants (0.76% of the analytic sample).

Vitality: Within the intrinsic-capacity framework, vitality has frequently been operationalized through nutritional status, although substantial heterogeneity exists in its measurement across studies [26]. The Chinese guideline for the assessment and maintenance of intrinsic capacity in older adults recommends nutritional screening within the vitality domain, including use of the Mini Nutritional Assessment–Short Form (MNA-SF) [27]. Because the CLHLS does not contain all items required to reconstruct the MNA-SF or another comprehensive nutritional assessment, BMI was used as an available anthropometric proxy for the nutritional component of vitality. The use of BMI as a vitality indicator has also been reported in previous intrinsic-capacity studies based on secondary or population-based data [28,29].

BMI was calculated from measured body weight and standing height. To retain an ordinal representation consistent with the BMI component of the MNA-SF, scores were assigned as follows: BMI < 19 kg/m^2^ = 0, 19 to <21 kg/m^2^ = 1, 21 to <23 kg/m^2^ = 2, and ≥23 kg/m^2^ = 3. Higher scores therefore indicate a lower level of undernutrition risk according to the BMI component of the MNA-SF rather than a comprehensive assessment of vitality or overall nutritional health. Participants with missing height or weight measurements were treated as having missing vitality data.

Psychological capacity: Psychological capacity was assessed using the CLHLS-adapted 10-item depressive-symptom measure. The CLHLS B3 module includes nine affective or cognitive symptom items referring primarily to the previous week, together with a survey-specific item assessing current sleep quality. Thus, this measure should not be interpreted as the standard CES-D-10 without modification. Responses were recoded according to the CLHLS-based scoring procedure so that higher total scores indicated greater depressive-symptom burden [30,31]. For the intrinsic-capacity analysis, the score was subsequently reversed so that higher values represented better psychological capacity. Participants with missing information required to calculate the psychological-capacity score were excluded from profile estimation.

Sensory function: Sensory function was operationalized using hearing and vision measures available in the CLHLS. Hearing was interviewer-rated according to whether the participant could hear the interviewer without a hearing aid, with a hearing aid, only partially with a hearing aid, or not at all. Vision was assessed using a ring-shaped optotype, and the CLHLS protocol specifically instructed participants to complete the test without eyeglasses. The vision indicator therefore reflects uncorrected visual performance rather than visual function under usual correction. Hearing and vision scores were combined to form a sensory-capacity score ranging from 0 to 4, with higher scores indicating better sensory function. This measure was therefore treated as a CLHLS-based proxy for the sensory domain of intrinsic capacity rather than as a direct implementation of the ICOPE sensory assessment.

#### 2.2.2. Unmet Needs for Home- and Community-Based Care Services

Eight home- and community-based care services were assessed using two corresponding sets of questions in the CLHLS questionnaire: whether each service was currently available in the participant’s community and whether the participant wanted the community to provide that service. The eight services were daily living assistance, home medical visits and medication delivery, emotional support or companionship, daily shopping assistance, social and recreational activities, legal assistance, health knowledge, and resolution of family or neighborhood disputes.

For each service, an unmet need was operationally defined as a participant reporting that they wanted the service while also reporting that the service was not currently available in their community. Such responses were coded as 1, whereas all other combinations were coded as 0. Thus, the measure represents a self-reported discrepancy between service preference and perceived local availability rather than an objectively assessed clinical or care need. Participants with missing information for any of the service items required to derive the unmet-need indicators were excluded from the corresponding latent-class analysis.

#### 2.2.3. Covariates

Covariates were grouped into five domains: sociodemographic characteristics, socioeconomic status, physical health status, intergenerational support from children, and social security. Sociodemographic characteristics included sex, age, marital status, educational attainment, place of residence (urban/rural), and living arrangement. Socioeconomic status included pre-retirement occupation and total annual household income. Physical health status included Basic Activities of Daily Living (BADL), self-rated health, and chronic disease status. BADL was assessed using six activities: eating, dressing, bathing, toileting, indoor mobility, and bladder/bowel control. Each activity was scored according to the level of assistance required (no assistance = 3, partial assistance = 2, and full assistance = 1), yielding a total score from 6 to 18; higher scores indicated greater self-care ability. Self-rated health was assessed on a five-point scale ranging from very poor to very good. Chronic disease status was based on 24 conditions, including hypertension and diabetes, and was categorized as no chronic disease, one chronic disease, or two or more chronic diseases. Intergenerational support included financial, emotional, and caregiving support. Financial support was measured as the total monetary value of gifts received from children, grandchildren, and spouses in the previous year. Emotional support was coded as 1 if the participant identified children, grandchildren, or a spouse as someone they usually talked with or confided in when troubled, and 0 otherwise. Caregiving support was coded as 1 if the participant identified children, grandchildren, or a spouse as someone who cared for them when unwell or whom they first turned to when facing difficulties, and 0 otherwise. Social security included pension insurance and medical insurance. Pension insurance was coded as 1 if the participant received a retirement pension, old-age pension, or commercial pension insurance, and 0 otherwise. Medical insurance was coded as 1 if the participant was covered by publicly funded medical care, urban employee or resident medical insurance, new rural cooperative medical insurance, or commercial medical insurance, and 0 otherwise.

### 2.3. Statistical Analysis

Descriptive analyses were based on observed available-case data. Continuous and ordinal variables were summarized as means with standard deviations or medians with interquartile ranges, as appropriate, and categorical variables were summarized as frequencies and percentages. Differences across latent subgroups were examined using the Kruskal–Wallis H test for non-normally distributed continuous or ordinal variables and Pearson’s chi-square test for categorical variables. Effect sizes were quantified using rank epsilon-squared (ε^2^) for Kruskal–Wallis tests and Cramér’s V for Pearson chi-square tests. All descriptive analyses were unweighted.

Intrinsic-capacity profiles were identified using a scale-aware finite-mixture model in Mplus. Because the five intrinsic-capacity domains differed in their measurement scales, cognition and psychological capacity were entered as standardized continuous indicators, whereas locomotion (0–6), vitality (0–3), and sensory capacity (0–4) were treated as ordinal indicators. Models with one through six latent classes were estimated using robust maximum likelihood estimation. To reduce the risk of local maxima, 5000 random sets of starting values were used, with 1000 retained for final-stage optimization and 20 initial-stage iterations. The optimal number of classes was determined by considering the Akaike information criterion (AIC), Bayesian information criterion (BIC), sample-size-adjusted BIC (aBIC), entropy, the adjusted Lo–Mendell–Rubin likelihood-ratio test (LMR-LRT), class size, replication of the best log-likelihood, parsimony, and substantive interpretability. Lower information-criterion values and higher entropy indicated better fit and classification quality. The bootstrap likelihood-ratio test was examined as a supplementary diagnostic but was not used as a primary criterion because numerical instability occurred in the four- versus five-class bootstrap comparison. Participants were assigned to their most likely class according to posterior probabilities. For graphical presentation only, domain scores were standardized to facilitate comparison across the five intrinsic-capacity domains.

Latent class analysis was used to identify patterns of unmet home- and community-based care needs based on the eight binary unmet-need indicators. One- through six-class models were fitted sequentially. Model selection was based on AIC, BIC, aBIC, entropy, the adjusted LMR-LRT, class size, solution stability, parsimony, and substantive interpretability. Participants were assigned to the pattern with the highest posterior probability.

The unadjusted association between intrinsic-capacity profiles and unmet-need patterns was first examined using cross-tabulation and Pearson’s chi-square test, with Cramér’s V used to quantify the magnitude of association. Because modal class assignment does not fully account for classification uncertainty, an additional sensitivity analysis repeated the cross-tabulation among participants with maximum posterior probabilities ≥ 0.80 for both the intrinsic-capacity profile and unmet-need pattern classifications.

Seven covariates contained missing data after sample screening: marital status, educational attainment, pre-retirement occupation, annual household income, financial support from children, emotional support from children, and caregiving support from children. Missing covariate data were handled using multiple imputation by fully conditional specification (FCS) in SPSS. The imputation model included the intrinsic-capacity profile, unmet-need pattern, and all sociodemographic, socioeconomic, social-security, intergenerational-support, and health-related variables subsequently considered in the regression analyses. Variables were specified according to their measurement level, with nominal and ordinal variables imputed within the corresponding categorical FCS framework. Financial support from children, treated as a continuous variable, was imputed using predictive mean matching with five nearest donors and constrained to non-negative values. The imputation procedure assumed that missingness was missing at random conditional on the variables included in the imputation model. Twenty imputed datasets were generated, with up to 20 FCS iterations per imputation. Twenty imputations were selected because the highest variable-level missing proportion was approximately 15%, providing adequate representation of imputation uncertainty. Multinomial logistic regression was performed separately within each imputed dataset, and regression coefficients and standard errors were subsequently combined using Rubin’s rules.

Multinomial logistic regression was used to examine adjusted associations between intrinsic-capacity profiles and unmet-need patterns, with the High-Need/Daily Care and Psychosocial Rights Pattern (Pattern 3) specified as the outcome reference category and the Comprehensive High-Functioning Profile (Class 5) as the intrinsic-capacity reference group. Covariates were selected a priori according to substantive knowledge and their presumed conceptual relationship with intrinsic capacity and unmet care needs rather than on the basis of univariate statistical significance. Model 1 adjusted for core sociodemographic and socioeconomic characteristics, including age, sex, marital status, residence, living arrangement, educational attainment, pre-retirement occupation, and annual household income. Model 2 additionally adjusted for pension and medical insurance and was specified as the primary confounder-adjusted model. Model 3 further included financial, emotional, and caregiving support from children, chronic disease status, BADL, and self-rated health. Because these contemporaneous health and support variables may partly reflect consequences, mediating pathways, or contextual responses to functional capacity rather than conventional confounders, Model 3 was treated as an extended adjustment analysis rather than the primary model. A classification-confidence sensitivity analysis repeated the primary Model 2 among participants with maximum posterior probabilities ≥ 0.80 for both latent classifications.

To assess potential selection bias arising from sample exclusions, characteristics of participants included in the final analytic sample (n = 10,405) were compared with those excluded during sample screening (n = 5469) using the original 2018 CLHLS records. Continuous and ordinal variables were compared using the Mann–Whitney U test, whereas categorical variables were compared using Pearson’s chi-square test. Analyses were based on available cases for each characteristic. Absolute standardized mean differences were calculated for continuous or ordinal variables, and Cramér’s V was used for categorical variables to describe the magnitude of between-group differences.

All analyses were conducted using SPSS version 25.0 and Mplus version 8.3, and figures were prepared in R version 4.4.3. Statistical tests were two-sided, with *p* < 0.05 considered statistically significant. Because the analyses were unweighted and focused on latent-pattern identification and associations rather than population prevalence estimation, descriptive proportions should not be interpreted as nationally representative prevalence estimates.

## 3. Results

### 3.1. Participant Characteristics and Comparison of Included and Excluded Participants

A total of 10,405 community-dwelling older adults were included in the final analytic sample. The median age was 83 years (Q1–Q3: 75–92), and 4906 participants (47.2%) were male. 

To assess potential selection bias, the 10,405 included participants were compared with the 5469 participants excluded during sample screening using the original 2018 CLHLS records (Supplementary Table S1). Excluded participants were older than those included [median 92 (81, 100) vs. 83 (75, 92) years; |SMD| = 0.568] and had poorer BADL performance [18 (12, 18) vs. 18 (18, 18); |SMD| = 0.771]. They were also less likely to be male (36.9% vs. 47.2%) or married (28.2% vs. 47.7%) and were more likely to be illiterate (63.0% vs. 43.8%). The overall effect sizes for marital status and educational attainment were Cramér’s V = 0.188 and 0.185, respectively. In contrast, differences in urban/rural residence (Cramér’s V = 0.003, *p* = 0.739) and pension insurance coverage (Cramér’s V = 0.005, *p* = 0.574) were minimal.

### 3.2. Intrinsic-Capacity Profiles

Scale-aware finite-mixture models with one through six classes were fitted to identify intrinsic-capacity profiles. Cognition and psychological capacity were modeled as continuous indicators, whereas locomotion, vitality, and sensory capacity were treated as ordinal indicators according to their original measurement scales. Model-fit statistics are presented in Table 1.

AIC, BIC, and aBIC decreased progressively as the number of classes increased. The five-class model had an entropy of 0.813, and the adjusted LMR test supported improvement over the four-class solution (*p* < 0.001). Although the six-class model yielded lower information-criterion values and slightly higher entropy (0.825), the adjusted LMR test did not support the addition of a sixth class (*p* = 0.771). Moreover, the six-class solution produced two relatively small classes (approximately 2.3% and 4.2%) and primarily subdivided existing impaired profiles rather than yielding a clearly distinct additional subgroup. All candidate models converged normally, and the best log-likelihood values were successfully replicated. Considering statistical fit, classification quality, class size, parsimony, solution stability, and substantive interpretability together, the five-class solution was retained.

**Figure 2:**
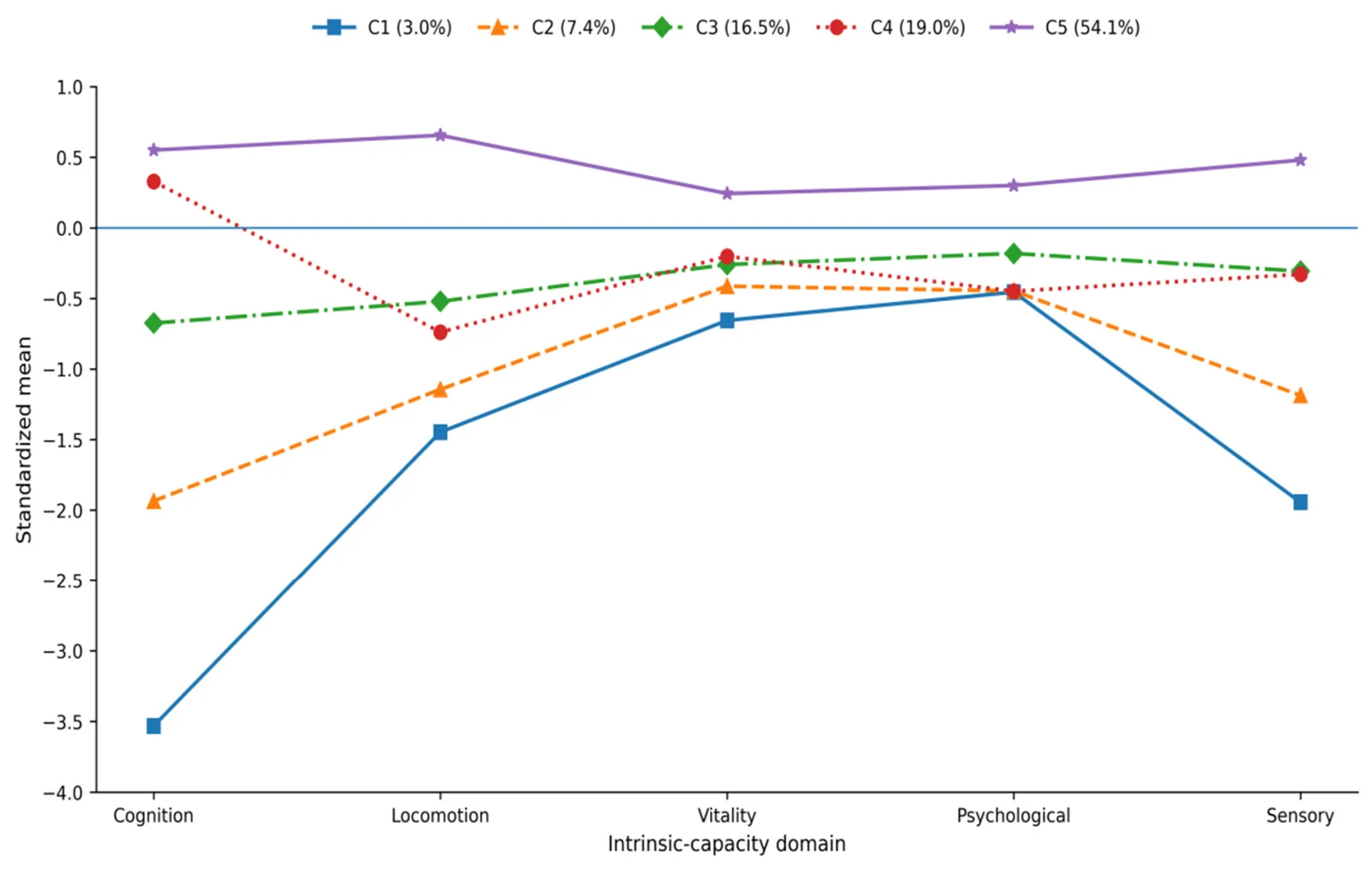
Standardized domain profiles of the final five intrinsic-capacity classes. Note: C1, Very Severe Multidomain Impairment Profile (n = 314, 3.0%); C2, Severe Multidomain Impairment Profile (n = 767, 7.4%); C3, Moderate Multidomain Vulnerability Profile (n = 1720, 16.5%); C4, Cognition-Preserved Locomotion–Psychological Vulnerability Profile (n = 1978, 19.0%); and C5, Comprehensive High-Functioning Profile (n = 5626, 54.1%). Higher standardized scores indicate relatively better intrinsic capacity in the corresponding domain.

The five intrinsic-capacity profiles are shown in Figure 2. Class 1 was labeled the Very Severe Multidomain Impairment Profile (n = 314, 3.0%) and was characterized by marked impairment across all five domains, particularly cognition and sensory capacity. Class 2, the Severe Multidomain Impairment Profile (n = 767, 7.4%), also showed impairment across multiple domains, although generally less severe than Class 1. Class 3, the Moderate Multidomain Vulnerability Profile (n = 1720, 16.5%), showed moderate reductions across several domains without the extreme deficits observed in Classes 1 and 2. Class 4, the Cognition-Preserved Locomotion–Psychological Vulnerability Profile (n = 1978, 19.0%), was distinguished by relatively preserved cognition together with lower locomotion and psychological capacity. Class 5, the Comprehensive High-Functioning Profile (n = 5626, 54.1%), showed relatively favorable levels across all five intrinsic-capacity domains.

Participant characteristics differed substantially across the five profiles (Table 2). In general, the more impaired profiles included older participants and greater proportions of women, divorced or widowed participants, and participants with lower educational attainment and poorer functional status, whereas the Comprehensive High-Functioning Profile was characterized by younger age and generally more favorable socioeconomic and health-related characteristics.

**Table 2:** Participant characteristics across intrinsic-capacity profiles.

Variable	Category	Overall n (%)/M (QL, QU) 10,405	Class 1 n (%)/M (QL, QU) 314 (3.0)	Class 2 n (%)/M (QL, QU) 767 (7.4)	Class 3 n (%)/M (QL, QU) 1720 (16.5)	Class 4 n (%)/M (QL, QU) 1978 (19.0)	Class 5 n (%)/M (QL, QU) 5626 (54.1)	H/χ^2^	*p*	Effect Size
Age (years)		83 (75, 92)	100 (94, 103)	96 (90, 101)	90 (83, 98)	88 (81, 94)	77 (71, 83)	3578.806	<0.001 ***	0.344
Gender	Male	4906 (47.2)	87 (27.7)	231 (30.1)	586 (34.1)	883 (44.6)	3119 (55.4)	415.148	<0.001 ***	0.200
	Female	5499 (52.8)	227 (72.3)	536 (69.9)	1134 (65.9)	1095 (55.4)	2507 (44.6)			
Marital status (n = 10,324)	Married	4922 (47.7)	25 (8.0)	127 (16.7)	483 (28.4)	706 (35.9)	3581 (64.1)	1471.728	<0.001 ***	0.267
	Divorced/Widowed	5323 (51.6)	285 (91.6)	626 (82.3)	1209 (71.0)	1243 (63.3)	1960 (35.1)			
	Unmarried	79 (0.8)	1 (0.3)	8 (1.1)	10 (0.6)	16 (0.8)	44 (0.8)			
Educational level (n = 8861)	Illiteracy	3881 (43.8)	221 (79.2)	506 (77.5)	1048 (70.7)	848 (49.9)	1258 (26.5)	1568.812	<0.001 ***	0.210
	Elementary school	3092 (34.9)	41 (14.7)	122 (18.7)	337 (22.7)	577 (34.0)	2015 (42.4)			
	Junior high	982 (11.1)	7 (2.5)	9 (1.4)	54 (3.6)	131 (7.7)	781 (16.4)			
	Secondary school	564 (6.4)	6 (2.2)	10 (1.5)	30 (2.0)	80 (4.7)	438 (9.2)			
	College or above	342 (3.9)	4 (1.4)	6 (0.9)	14 (0.9)	62 (3.7)	256 (5.4)			
Place of residence	City	5765 (55.4)	163 (51.9)	392 (51.1)	890 (51.7)	1121 (56.7)	3199 (56.9)	22.727	<0.001 ***	0.047
	Rural	4640 (44.6)	151 (48.1)	375 (48.9)	830 (48.3)	857 (43.3)	2427 (43.1)			
Residence arrangement	Living alone	1831 (17.6)	43 (13.7)	122 (15.9)	350 (20.3)	394 (19.9)	922 (16.4)	26.817	<0.001 ***	0.051
	Not living alone	8574 (82.4)	271 (86.3)	645 (84.1)	1370 (79.7)	1584 (80.1)	4704 (83.6)			
Occupation before retirement (n = 8827)	Technical/management	1053 (11.9)	16 (5.8)	26 (4.0)	65 (4.4)	195 (11.5)	751 (15.9)	319.195	<0.001 ***	0.134
	General employees	1345 (15.2)	30 (10.8)	65 (10.0)	145 (9.8)	266 (15.7)	839 (17.7)			
	Farmers/others	6429 (72.8)	232 (83.5)	556 (85.9)	1270 (85.8)	1229 (72.7)	3142 (66.4)			
Household annual total income (RMB) (n = 9657)	≤50,000	6433 (66.6)	214 (74.6)	521 (74.4)	1090 (71.1)	1210 (67.0)	3398 (63.7)	67.824	<0.001 ***	0.059
	50,001–99,999	1334 (13.8)	33 (11.5)	88 (12.6)	190 (12.4)	228 (12.6)	795 (14.9)			
	≥100,000	1890 (19.6)	40 (13.9)	91 (13.0)	253 (16.5)	367 (20.3)	1139 (21.4)			
Pension insurance	No	5236 (50.3)	189 (60.2)	482 (62.8)	951 (55.3)	1027 (51.9)	2587 (46.0)	121.709	<0.001 ***	0.108
	Yes	5169 (49.7)	125 (39.8)	285 (37.2)	769 (44.7)	951 (48.1)	3039 (54.0)			
Medical insurance	No	1141 (11.0)	51 (16.2)	119 (15.5)	203 (11.8)	207 (10.5)	561 (10.0)	32.648	<0.001 ***	0.056
	Yes	9264 (89.0)	263 (83.8)	648 (84.5)	1517 (88.2)	1771 (89.5)	5065 (90.0)			
Financial support from children (1000 RMB) (n = 9795)		1.6 (0, 4.0)	1.0 (0, 2.9)	1.0 (0, 3.0)	1.5 (0.3, 3.5)	1.6 (0, 4.0)	2.0 (0, 4.0)	37.672	<0.001 ***	0.004
Emotional support from children (n = 10,389)	No	1483 (14.3)	20 (6.5)	61 (8.0)	208 (12.1)	224 (11.3)	970 (17.3)	101.320	<0.001 ***	0.099
	Yes	8906 (85.7)	290 (93.5)	703 (92.0)	1510 (87.9)	1750 (88.7)	4653 (82.7)			
Care support from children (n = 10,404)	No	1015 (9.8)	6 (1.9)	38 (5.0)	121 (7.0)	144 (7.3)	706 (12.5)	120.072	<0.001 ***	0.107
	Yes	9389 (90.2)	308 (98.1)	729 (95.0)	1599 (93.0)	1833 (92.7)	4920 (87.5)			
BADL score		18 (18, 18)	16 (12, 18)	18 (15, 18)	18 (17, 18)	18 (18, 18)	18 (18, 18)	2198.065	<0.001 ***	0.211
Self-rated health		3 (3, 4)	3 (3, 4)	3 (3, 4)	3 (3, 4)	3 (3, 4)	4 (3, 4)	409.261	<0.001 ***	0.039
Chronic disease	None	3100 (29.8)	142 (45.2)	265 (34.6)	548 (31.9)	513 (25.9)	1632 (29.0)	94.722	<0.001 ***	0.067
	Single	3304 (31.8)	85 (27.1)	249 (32.5)	548 (31.9)	572 (28.9)	1850 (32.9)			
	≥2 conditions	4001 (38.5)	87 (27.7)	253 (33.0)	624 (36.3)	893 (45.1)	2144 (38.1)			

Note: Descriptive statistics are unweighted and based on observed available-case data; multiple imputation was used for the regression analyses rather than for this descriptive table. Data are presented as n (%) for categorical variables and median (lower quartile, upper quartile), M (QL, QU), for continuous/ordinal variables. Valid sample sizes are shown for variables with missing observations. Financial support from children is expressed in units of 1000 RMB. H denotes the Kruskal–Wallis H statistic and χ^2^ denotes the Pearson chi-square statistic. Effect sizes are reported as rank epsilon-squared (ε^2^) for variables analyzed using the Kruskal–Wallis test and Cramér’s V for categorical variables analyzed using the Pearson chi-square test. Class 1 = Very Severe Multidomain Impairment Profile; Class 2 = Severe Multidomain Impairment Profile; Class 3 = Moderate Multidomain Vulnerability Profile; Class 4 = Cognition-Preserved Locomotion-Psychological Vulnerability Profile; Class 5 = Comprehensive High-Functioning Profile. *** *p* < 0.001.

### 3.3. Latent Patterns of Unmet Home- and Community-Based Care Needs

Latent class models with one through six classes were estimated for the eight unmet home- and community-based care needs. Model-fit statistics are presented in Table 3. AIC, BIC, and aBIC decreased with increasing numbers of classes. The three-class model had an entropy of 0.826 and a significant adjusted LMR test relative to the two-class solution (*p* < 0.001). Although the four-class model yielded lower information-criterion values, its entropy was slightly lower (0.808), and the additional class primarily subdivided an existing heterogeneous pattern without producing a clearly distinct and substantively meaningful unmet-need configuration. Models with five and six classes showed further reductions in entropy. Considering fit, classification quality, class balance, parsimony, solution stability, and substantive interpretability, the three-class solution was retained.

**Figure 3:**
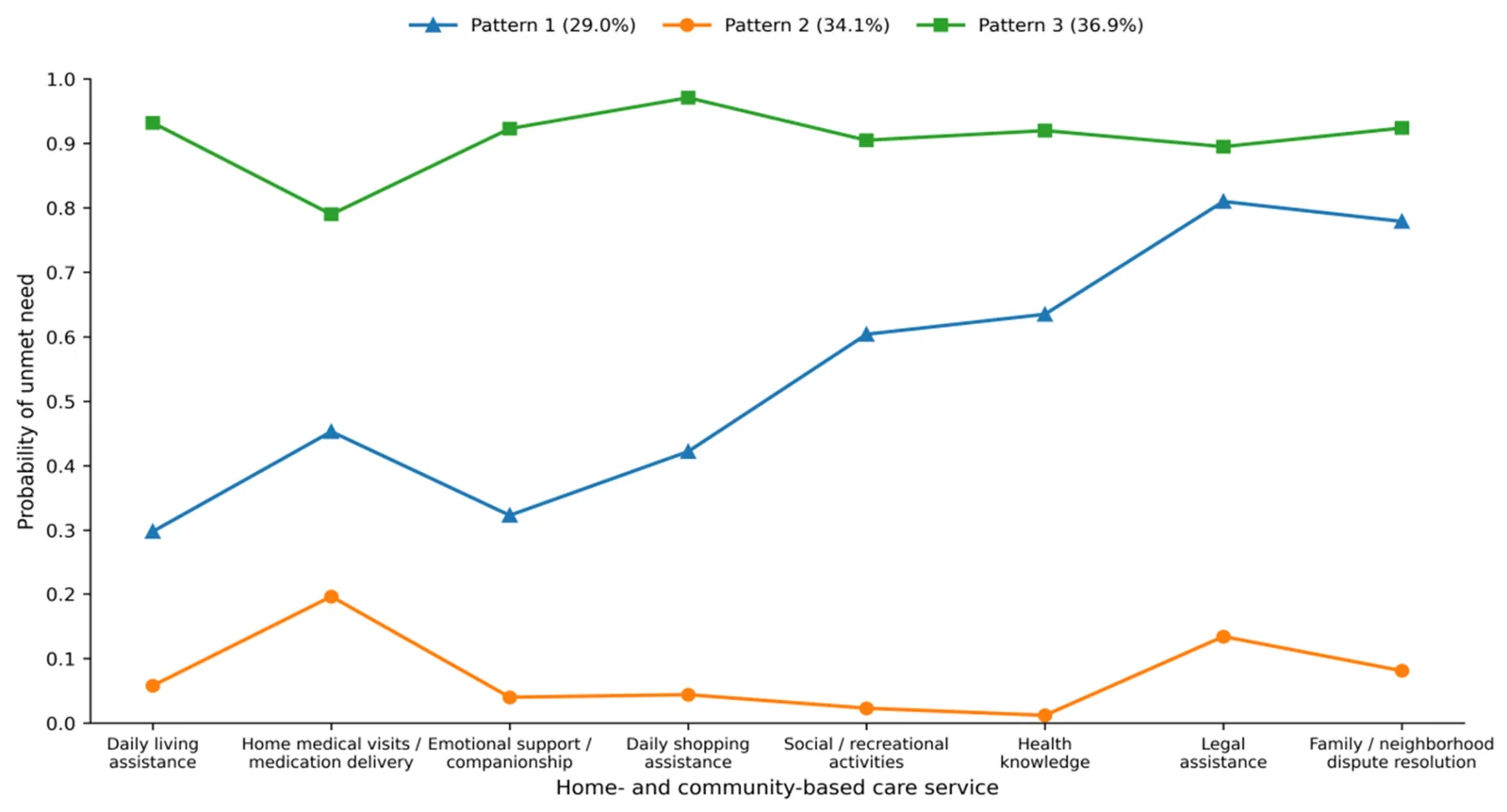
Item-response probability profiles of the final three unmet-need patterns for home- and community-based care services. Note: Patterns are presented in the final manuscript order: Pattern 1 = Rights and Health Dual-Unmet Need Pattern (n = 3017, 29.0%); Pattern 2 = Low-Need/Light Health Protection Pattern (n = 3553, 34.1%); and Pattern 3 = High-Need/Daily Care and Psychosocial Rights Pattern (n = 3835, 36.9%). Higher probabilities indicate a greater likelihood of reporting an unmet need for the corresponding service item.

The conditional probabilities of the eight unmet needs across the three patterns are shown in Figure 3. Pattern 1, the Rights and Health Dual-Unmet Need Pattern (n = 3017, 29.0%), was characterized by relatively high probabilities of unmet legal assistance (0.810), family/neighborhood dispute resolution (0.779), health knowledge (0.635), and social/recreational activity needs (0.604), together with moderate probabilities for several daily and medical services. Pattern 2, the Low-Need/Light Health Protection Pattern (n = 3553, 34.1%), showed generally low probabilities across all eight unmet-need items, with home medical visits/medication delivery representing the most prominent unmet need (0.196). Pattern 3, the High-Need/Daily Care and Psychosocial Rights Pattern (n = 3835, 36.9%), showed consistently high probabilities across all eight services, ranging from approximately 0.79 to 0.97.

Characteristics of participants across the three unmet-need patterns are shown in Table 4. Age and sex did not differ significantly across patterns (*p* = 0.736 and *p* = 0.619, respectively), whereas marital status, residence, living arrangement, educational attainment, occupation, household income, social-security coverage, intergenerational support, self-rated health, and chronic-disease status showed significant between-pattern differences. Despite statistical significance for several characteristics, the corresponding effect sizes were generally small.

### 3.4. Unadjusted Association Between Intrinsic-Capacity Profiles and Unmet-Need Patterns

The overall unadjusted distribution of unmet-need patterns did not differ significantly across intrinsic-capacity profiles (χ^2^(8) = 14.77, *p* = 0.064; Cramér’s V = 0.027), indicating that the crude association between the two latent classifications was very small.

Because assignment to latent classes was based on maximum posterior probability, a sensitivity analysis was conducted among participants with high classification confidence for both latent solutions. Restricting the analysis to participants with maximum posterior probabilities ≥ 0.80 for both the intrinsic-capacity and unmet-need classifications yielded a similar result (n = 6706; χ^2^(8) = 12.26, *p* = 0.140; Cramér’s V = 0.030). Thus, the absence of a strong overall crude association was not explained by participants with lower classification certainty.

### 3.5. Adjusted Associations Between Intrinsic-Capacity Profiles and Unmet-Need Patterns

Multinomial logistic regression was used to examine the associations between intrinsic-capacity profiles and unmet-need patterns. Pattern 3 (High-Need/Daily Care and Psychosocial Rights Pattern) served as the outcome reference category, and Class 5 (Comprehensive High-Functioning Profile) served as the intrinsic-capacity reference group. Estimates from the primary confounder-adjusted Model 2 are presented in Table 5, whereas Model 1, the extended Model 3, and the classification-confidence sensitivity analysis are provided in Supplementary Table S2.

In the primary Model 2, participants in the Very Severe Multidomain Impairment Profile (Class 1) had higher odds of belonging to the Rights and Health Dual-Unmet Need Pattern rather than the High-Need/Daily Care and Psychosocial Rights Pattern compared with those in the Comprehensive High-Functioning Profile (OR = 1.40, 95% CI 1.02–1.92, *p* = 0.035). Participants in the Cognition-Preserved Locomotion–Psychological Vulnerability Profile (Class 4) also had higher odds of Pattern 1 versus Pattern 3 (OR = 1.30, 95% CI 1.13–1.49, *p* < 0.001). Neither the Severe Multidomain Impairment Profile nor the Moderate Multidomain Vulnerability Profile was significantly associated with Pattern 1 versus Pattern 3.

No intrinsic-capacity profile was significantly associated with the Low-Need/Light Health Protection Pattern versus the High-Need/Daily Care and Psychosocial Rights Pattern in the primary model. The ORs for Classes 1–4 versus Class 5 were 1.32 (95% CI 0.98–1.77), 1.06 (95% CI 0.87–1.30), 1.01 (95% CI 0.87–1.17), and 1.07 (95% CI 0.94–1.23), respectively.

In the extended Model 3, which additionally adjusted for intergenerational-support and health-related characteristics, the association for the Very Severe Multidomain Impairment Profile was attenuated and was no longer statistically significant (OR = 1.27, 95% CI 0.91–1.78, *p* = 0.156), whereas the association for the Cognition-Preserved Locomotion–Psychological Vulnerability Profile remained significant (OR = 1.21, 95% CI 1.05–1.39, *p* = 0.010).

The classification-confidence sensitivity analysis, restricted to participants with maximum posterior probabilities ≥ 0.80 for both latent classifications, showed a similar pattern. In this analysis, Class 1 (OR = 1.80, 95% CI 1.24–2.62, *p* = 0.002) and Class 4 (OR = 1.44, 95% CI 1.16–1.80, *p* = 0.001) were both associated with higher odds of Pattern 1 versus Pattern 3.

## 4. Discussion

Using data from a large sample of community-dwelling older adults in China, this study identified five distinct intrinsic-capacity profiles and three patterns of unmet home- and community-based care needs. Three findings are particularly noteworthy. First, intrinsic capacity was heterogeneous: more than half of the participants belonged to the Comprehensive High-Functioning Profile, whereas the remaining participants were distributed across profiles characterized by varying degrees and combinations of multidomain vulnerability. Second, unmet service needs also clustered into distinct patterns rather than occurring independently, including a Rights and Health Dual-Unmet Need Pattern, a Low-Need/Light Health Protection Pattern, and a High-Need/Daily Care and Psychosocial Rights Pattern. Third, the overall crude association between intrinsic-capacity profiles and unmet-need patterns was very small and did not reach statistical significance.

The identified profiles and unmet-need patterns further highlight the multidimensional heterogeneity of both functional capacity and service needs among older adults. Intrinsic capacity did not follow a simple continuum from “healthy” to “impaired”; instead, some profiles showed broad multidomain impairment, whereas others combined preserved and vulnerable capacities, consistent with the multidimensional WHO intrinsic-capacity framework [10,11]. Likewise, unmet needs ranged from generally low levels to selective rights- and health-related needs and broadly elevated needs across multiple service domains. Together, these findings suggest that intrinsic capacity alone is insufficient to explain unmet service needs, which are also likely shaped by socioeconomic resources, family support, health conditions, and the service environment.

Although the overall crude association between intrinsic-capacity profiles and unmet-need patterns was very small and not statistically significant, the primary confounder-adjusted model identified associations of both the Very Severe Multidomain Impairment Profile and the Cognition-Preserved Locomotion–Psychological Vulnerability Profile with Pattern 1 versus Pattern 3. Importantly, Pattern 3 showed consistently higher probabilities of unmet need than Pattern 1 across all eight service items. Therefore, the Pattern 1 versus Pattern 3 contrast should not be interpreted as indicating greater absolute unmet need for rights-, health-, or social-participation services. Rather, Pattern 1 represents a more selective configuration of unmet needs, in which rights-related, health-information, dispute-resolution, and social-participation needs were relatively prominent within the pattern, whereas Pattern 3 was characterized by broadly elevated unmet needs across daily care, medical, psychosocial, and rights-related services. Accordingly, the association observed for the Cognition-Preserved Locomotion–Psychological Vulnerability Profile indicates a greater relative likelihood of belonging to a selective rather than a uniformly elevated unmet-need pattern. This distinction is important because the multinomial model compares relative pattern membership and does not demonstrate that participants in Pattern 1 had higher absolute levels of any specific unmet need than those in Pattern 3. One possible interpretation is that relatively preserved cognition may be compatible with a more differentiated pattern of perceived service needs, whereas reduced locomotion and psychological capacity may contribute to unmet needs in selected domains without necessarily producing uniformly high unmet need across all services. However, this interpretation remains speculative because the present study did not directly assess processes of need recognition, preference formation, or help-seeking. Longitudinal studies incorporating these constructs are needed to clarify why particular intrinsic-capacity profiles may be associated with selective rather than broadly elevated unmet-need configurations.

The association for the Very Severe Multidomain Impairment Profile was less stable across analyses. It was nominally significant in Model 1 (OR = 1.37, *p* = 0.049) and the primary Model 2 (OR = 1.40, *p* = 0.035), but was attenuated after additional adjustment for contemporaneous family-support and health-related characteristics in Model 3 (OR = 1.27, *p* = 0.156). Conversely, the estimate became larger in the high-classification-confidence sensitivity analysis (OR = 1.80, *p* = 0.002). Given the relatively small size of this profile (n = 314), these variations indicate sensitivity to model specification and sample restriction. The Class 1 association should therefore be regarded as exploratory rather than as a robust profile-specific finding. More generally, because multiple profile contrasts were examined against a non-significant overall crude association, individual regression coefficients should be interpreted cautiously. Similarly, no intrinsic-capacity profile was independently associated with the Low-Need/Light Health Protection Pattern versus the High-Need/Daily Care and Psychosocial Rights Pattern in either the primary or extended adjusted model. A lower level of reported unmet need should therefore not be interpreted simply as better intrinsic capacity, as it may also reflect differences in perceived demand, informal support, service availability, or access barriers. Together, these findings reinforce the need to consider intrinsic capacity alongside social, economic, family, and health-related factors when assessing unmet care needs.

The selection-related differences should also be considered when interpreting these findings. Participants excluded during sample screening were substantially older and had poorer BADL performance than those included in the analytic sample, with relatively large standardized differences for age and BADL. This suggests that the final analytic sample may underrepresent the oldest and most functionally impaired segments of the population. Accordingly, the observed profile distributions and associations may not fully reflect those of the frailest older adults and should be interpreted in the context of this potential selection bias.

The findings have several implications for home- and community-based care planning. First, intrinsic-capacity assessment may help identify functionally distinct subgroups that could benefit from different priorities in further needs assessment. However, the present results do not support assigning fixed service packages solely on the basis of intrinsic-capacity profile membership. Instead, profile information may be most useful as an initial screening layer that is combined with direct assessment of service needs and preferences, socioeconomic resources, family support, health status, and locally available services. Second, the identification of distinct unmet-need patterns suggests that service planning may benefit from coordinated bundles of related services rather than isolated interventions. For example, Pattern 1 represented a selective configuration in which rights-related, health-information, dispute-resolution, and social-participation needs were relatively prominent, whereas Pattern 3 was characterized by broadly elevated unmet needs across all service domains. Such pattern information may assist community care providers in prioritizing comprehensive assessment and coordinating services, but prospective studies are needed before profile-based allocation strategies can be recommended. At the national level, these findings may also inform broader healthy ageing and long-term care planning by supporting the integration of multidimensional intrinsic-capacity assessment with direct assessment of service needs. National policy frameworks could provide common principles for needs assessment and service coordination, while allowing local implementation to be adapted to regional differences in population characteristics, resources, and service availability.

This study has several strengths. It used a large community-based sample and simultaneously examined multidimensional intrinsic capacity and multiple unmet home- and community-based care needs. The mixture-model specification accounted for differences in measurement scale across intrinsic-capacity domains by treating continuous and ordinal indicators appropriately. Model selection considered statistical fit, classification quality, class size, solution stability, parsimony, and substantive interpretability rather than relying on a single fit statistic. Missing covariate data were addressed using multiple imputation with Rubin’s-rule pooling, and sensitivity analyses incorporating classification confidence were performed. In addition, characteristics of included and excluded participants were compared to evaluate potential selection-related differences.

Several limitations should nevertheless be considered. First, the cross-sectional design precludes conclusions regarding temporal or causal relationships between intrinsic capacity and unmet service needs. Longitudinal studies are required to determine whether changes in intrinsic capacity precede changes in unmet needs or whether unmet support needs themselves contribute to subsequent functional decline. Second, latent-profile and latent-class assignments are probabilistic rather than directly observed categories. Although classification quality was acceptable and a high-confidence sensitivity analysis yielded a similar overall conclusion, some classification uncertainty remains. In addition, although the five-class solution was retained based on the adjusted LMR test, solution stability, and substantive interpretability, its entropy (0.813) was lower than that of the four-class solution (0.922), reflecting a trade-off between greater profile differentiation and classification precision. Third, participants excluded because of missing key information differed from those included in several characteristics, particularly age, BADL, marital status, and educational attainment. The analytic sample may therefore underrepresent very old adults, those with poorer functional status, or those with greater socioeconomic vulnerability, and selection bias cannot be fully excluded. Fourth, the analyses were unweighted. Because the primary objective was to identify latent patterns and examine associations rather than estimate population prevalence, the reported class proportions and descriptive estimates should not be interpreted as nationally representative prevalence estimates. Fifth, unmet needs were measured using a fixed set of eight home- and community-based care services available in the CLHLS questionnaire. In addition, these indicators were derived from participants’ reported service preferences and perceived community availability rather than from an objective assessment of clinical or care needs. They should therefore be interpreted as preference–availability gaps, which may also be influenced by service awareness, expectations, and local context. Other potentially important needs, such as rehabilitation, home modification, transportation, digital support, and more detailed mental-health services, were not assessed. Sixth, province- and community-level clustering and regional fixed effects were not explicitly modeled, and detailed contextual information on local service environments was unavailable; therefore, residual geographic heterogeneity may remain, and future multilevel longitudinal studies integrating both demand- and supply-side factors are warranted. Seventh, although questionnaire-coded inability to answer was distinguished from true missingness in the cognitive assessment, the conservative assignment of the lowest score to isolated missing locomotion items may have modestly underestimated locomotor capacity for a small number of participants if the missing response was unrelated to actual performance. In addition, vitality was operationalized using the BMI component of nutritional assessment because the CLHLS did not contain sufficient information to reconstruct the full MNA-SF or another comprehensive vitality measure. BMI does not capture important aspects of vitality such as recent weight loss, appetite, muscle strength, body composition, or metabolic reserve; therefore, this indicator should be interpreted as a proxy for the nutritional component of vitality rather than a comprehensive vitality measure. Finally, the analysis was based on the 2018 wave of the CLHLS. Given subsequent changes in China’s population structure, home- and community-based care policies, and local service availability, the unmet-need patterns observed in this study may not fully reflect the current service environment. Future studies using more recent data are warranted to examine the temporal stability and contemporary applicability of these findings.

## 5. Conclusions

This study identified five distinct intrinsic-capacity profiles and three patterns of unmet home- and community-based care needs among community-dwelling older adults in China. The overall association between intrinsic-capacity profiles and unmet-need patterns was weak and not statistically significant in the crude analysis. In adjusted analyses, the Cognition-Preserved Locomotion–Psychological Vulnerability Profile showed the more consistent association with a selective rather than uniformly elevated unmet-need pattern, whereas the association for the Very Severe Multidomain Impairment Profile varied across model specifications and should be considered exploratory. These findings indicate that intrinsic capacity may contribute to understanding heterogeneity in service needs but should not be used alone to determine service allocation. Integrating intrinsic-capacity assessment with direct assessment of unmet needs, socioeconomic circumstances, family support, health status, and service availability may provide a more appropriate basis for person-centered home- and community-based care planning. At the national level, such an approach may also inform broader policy frameworks for needs assessment and service coordination, while allowing implementation to remain responsive to regional and local contexts.

## Figures and Tables

**Table 1:** Comparison of fitting indexes of latent profile models with different categories.

No. of Classes	AIC	BIC	aBIC	Entropy	LMR *p*	Class Proportion (%)	Smallest Class (%)
1	139,516.573	139,639.823	139,585.800	—	—	100.0	—
2	129,932.517	130,171.769	130,066.899	0.938	<0.001	86.4/13.6	13.6
3	126,444.606	126,799.858	126,644.143	0.925	<0.001	5.8/17.3/77.0	5.8
4	124,589.051	125,060.303	124,853.742	0.922	<0.001	70.5/18.4/3.2/8.0	3.2
5	123,512.615	124,099.868	123,842.461	0.813	<0.001	3.0/7.4/16.5/19.0/54.1	3.0
6	122,585.177	123,288.431	122,980.178	0.825	0.771	16.9/2.3/4.2/51.2/17.2/8.2	2.3

Note: AIC, Akaike information criterion; BIC, Bayesian information criterion; aBIC, sample-size adjusted Bayesian information criterion; LMR, Lo–Mendell–Rubin adjusted likelihood ratio test. The five-class model was retained as the final solution based on information criteria, entropy, class size, solution stability, significant adjusted LMR compared with the four-class model, and substantive interpretability. Although the six-class model showed lower information criteria, the adjusted LMR test was not significant and the solution yielded smaller and more fragmented classes. BLRT was not presented in the main table because the four- versus five-class comparison showed numerical instability in TECH14 bootstrap estimation. Class proportions may not sum to exactly 100% because of rounding.

**Table 3:** Comparison of fitting indexes of latent class models for unmet-need patterns.

No. of Classes	AIC	BIC	aBIC	Entropy	LMR *p*	Class Proportion (%)	Smallest Class (%)
1	114,271.085	114,329.086	114,303.663	—	—	100.0	—
2	83,964.658	84,087.908	84,033.885	0.916	<0.001	42.2/57.8	42.2
3	80,338.462	80,526.963	80,444.339	0.826	<0.001	29.0/34.1/36.9	29.0
4	79,049.137	79,302.889	79,191.664	0.808	<0.001	11.1/19.9/34.0/35.0	11.1
5	78,632.641	78,951.643	78,811.817	0.752	<0.001	14.0/25.4/14.9/16.6/29.1	14.0
6	78,478.624	78,862.877	78,694.450	0.735	0.003	20.6/3.3/15.1/25.5/12.2/23.3	3.3

Note: AIC, Akaike information criterion; BIC, Bayesian information criterion; aBIC, sample-size adjusted Bayesian information criterion; LMR, Lo–Mendell–Rubin adjusted likelihood ratio test. Class proportions are based on most-likely class membership. The three-class solution was retained after considering fit indices, entropy, class balance, parsimony, solution stability, and substantive interpretability. The four-class solution mainly subdivided an existing heterogeneous class without yielding a clearly distinct additional pattern.

**Table 4:** Participant characteristics across unmet-need patterns.

Variable	Category	Overall n (%)/M (QL, QU) 10,405	Pattern 1 n (%)/M (QL, QU) 3017 (29.0)	Pattern 2 n (%)/M (QL, QU) 3553 (34.1)	Pattern 3 n (%)/M (QL, QU) 3835 (36.9)	H/χ^2^	*p*	Effect Size
Age (years)		83 (75, 92)	83 (75, 92)	82 (74, 92)	83 (75, 92)	0.612	0.736	<0.001
Gender	Male	4906 (47.2)	1445 (47.9)	1662 (46.8)	1799 (46.9)	0.959	0.619	0.010
	Female	5499 (52.8)	1572 (52.1)	1891 (53.2)	2036 (53.1)			
Marital status (n = 10,324)	Married	4922 (47.7)	1490 (49.6)	1630 (46.4)	1802 (47.3)	11.477	0.022 *	0.024
	Divorced/Widowed	5323 (51.6)	1492 (49.7)	1850 (52.6)	1981 (52.0)			
	Unmarried	79 (0.8)	20 (0.7)	36 (1.0)	23 (0.6)			
Educational level (n = 8861)	Illiteracy	3881 (43.8)	1123 (43.1)	1369 (46.1)	1389 (42.3)	29.888	<0.001 ***	0.041
	Elementary school	3092 (34.9)	902 (34.6)	1032 (34.7)	1158 (35.3)			
	Junior high	982 (11.1)	287 (11.0)	316 (10.6)	379 (11.5)			
	Secondary school	564 (6.4)	161 (6.2)	174 (5.9)	229 (7.0)			
	College or above	342 (3.9)	132 (5.1)	81 (2.7)	129 (3.9)			
Place of residence	City	5765 (55.4)	1824 (60.5)	1862 (52.4)	2079 (54.2)	46.312	<0.001 ***	0.067
	Rural	4640 (44.6)	1193 (39.5)	1691 (47.6)	1756 (45.8)			
Residence arrangement	Living alone	1831 (17.6)	489 (16.2)	681 (19.2)	661 (17.2)	10.397	0.006 **	0.032
	Not living alone	8574 (82.4)	2528 (83.8)	2872 (80.8)	3174 (82.8)			
Occupation before retirement (n = 8827)	Technical/management	1053 (11.9)	316 (12.2)	279 (9.4)	458 (14.0)	81.910	<0.001 ***	0.068
	General employees	1345 (15.2)	446 (17.2)	352 (11.9)	547 (16.7)			
	Farmers/others	6429 (72.8)	1834 (70.6)	2330 (78.7)	2265 (69.3)			
Household annual total income (RMB) (n = 9657)	≤50,000	6433 (66.6)	1688 (60.5)	2416 (73.9)	2329 (64.7)	141.890	<0.001 ***	0.086
	50,001–99,999	1334 (13.8)	494 (17.7)	349 (10.7)	491 (13.6)			
	≥100,000	1890 (19.6)	606 (21.7)	503 (15.4)	781 (21.7)			
Pension insurance	No	5236 (50.3)	1351 (44.8)	1988 (56.0)	1897 (49.5)	83.259	<0.001 ***	0.089
	Yes	5169 (49.7)	1666 (55.2)	1565 (44.0)	1938 (50.5)			
Medical insurance	No	1141 (11.0)	274 (9.1)	390 (11.0)	477 (12.4)	19.482	<0.001 ***	0.043
	Yes	9264 (89.0)	2743 (90.9)	3163 (89.0)	3358 (87.6)			
Financial support from children (1000 RMB) (n = 9795)		1.6 (0, 4.0)	2.0 (0.3, 5.0)	1.4 (0, 3.0)	1.5 (0, 4.0)	87.312	<0.001 ***	0.009
Emotional support from children (n = 10,389)	No	1483 (14.3)	374 (12.4)	586 (16.5)	523 (13.7)	24.596	<0.001 ***	0.049
	Yes	8906 (85.7)	2643 (87.6)	2959 (83.5)	3304 (86.3)			
Care support from children (n = 10,404)	No	1015 (9.8)	248 (8.2)	377 (10.6)	390 (10.2)	11.787	0.003 **	0.034
	Yes	9389 (90.2)	2769 (91.8)	3176 (89.4)	3444 (89.8)			
BADL score		18 (18, 18)	18 (18, 18)	18 (18, 18)	18 (18, 18)	8.683	0.013 *	0.001
Self-rated health		3 (3, 4)	3 (3, 4)	3 (3, 4)	4 (3, 4)	46.970	<0.001 ***	0.005
Chronic disease	None	3100 (29.8)	801 (26.5)	1188 (33.4)	1111 (29.0)	63.478	<0.001 ***	0.055
	Single	3304 (31.8)	925 (30.7)	1159 (32.6)	1220 (31.8)			
	≥2 conditions	4001 (38.5)	1291 (42.8)	1206 (33.9)	1504 (39.2)			

Note: Descriptive statistics are unweighted and based on observed available-case data; multiple imputation was used for the regression analyses rather than for this descriptive table. Data are presented as n (%) for categorical variables and median (lower quartile, upper quartile), M (QL, QU), for continuous/ordinal variables. Valid sample sizes are shown for variables with missing observations. Financial support from children is expressed in units of 1000 RMB. H denotes the Kruskal–Wallis H statistic and χ^2^ denotes the Pearson chi-square statistic. Effect sizes are reported as rank epsilon-squared (ε^2^) for variables analyzed using the Kruskal–Wallis test and Cramér’s V for categorical variables analyzed using the Pearson chi-square test. Pattern 1 = Rights and Health Dual-Unmet Need Pattern; Pattern 2 = Low-Need/Light Health Protection Pattern; Pattern 3 = High-Need/Daily Care and Psychosocial Rights Pattern. * *p* < 0.05, ** *p* < 0.01, *** *p* < 0.001.

**Table 5:** Rubin-pooled multinomial logistic regression of intrinsic-capacity profiles and unmet-need patterns (primary Model 2).

Variable	Category/Contrast	Pattern 1 vs. Pattern 3 OR (95% CI)	*p*	Pattern 2 vs. Pattern 3 OR (95% CI)	*p*
Intrinsic capacity profile	Class 1: Very Severe Multidomain Impairment	1.402 (1.024–1.920)	0.035 *	1.320 (0.983–1.774)	0.065
	Class 2: Severe Multidomain Impairment	1.150 (0.926–1.427)	0.207	1.063 (0.867–1.303)	0.559
	Class 3: Moderate Multidomain Vulnerability	1.088 (0.932–1.271)	0.287	1.008 (0.870–1.168)	0.913
	Class 4: Cognition-Preserved Locomotion–Psychological Vulnerability	1.299 (1.131–1.492)	<0.001 ***	1.072 (0.937–1.228)	0.312
	Class 5: Comprehensive High-Functioning	Reference	—	Reference	—
Age	Per 1-year increase	0.995 (0.989–1.001)	0.136	0.996 (0.990–1.002)	0.148
Sex	Male vs. Female	1.090 (0.976–1.217)	0.125	1.078 (0.969–1.199)	0.167
Marital status	Married vs. Unmarried	0.988 (0.535–1.826)	0.969	0.696 (0.407–1.192)	0.187
	Divorced/Widowed vs. Unmarried	0.885 (0.479–1.634)	0.696	0.710 (0.415–1.214)	0.210
Residence	Urban vs. Rural	1.339 (1.204–1.490)	<0.001 ***	1.157 (1.046–1.279)	0.004 **
Living arrangement	Not living alone vs. Living alone	0.987 (0.855–1.140)	0.862	0.958 (0.839–1.095)	0.532
Education	Illiteracy vs. College or above	0.799 (0.578–1.106)	0.176	0.942 (0.668–1.327)	0.732
	Elementary school vs. College or above	0.706 (0.520–0.958)	0.025 *	0.902 (0.648–1.255)	0.540
	Junior high vs. College or above	0.686 (0.502–0.939)	0.019 *	1.005 (0.719–1.406)	0.976
	Secondary school vs. College or above	0.668 (0.482–0.926)	0.015 *	1.071 (0.758–1.511)	0.698
Occupation before retirement	Technical/management vs. Farmers/others	0.628 (0.503–0.783)	<0.001 ***	0.608 (0.490–0.754)	<0.001 ***
	General employees vs. Farmers/others	0.787 (0.667–0.929)	0.005 **	0.642 (0.541–0.761)	<0.001 ***
Household annual income	≤50,000 RMB vs. ≥100,000 RMB	0.929 (0.810–1.067)	0.297	1.399 (1.217–1.609)	<0.001 ***
	50,001–99,999 RMB vs. ≥100,000 RMB	1.271 (1.073–1.504)	0.005 **	1.078 (0.901–1.291)	0.411
Pension insurance	No vs. Yes	0.798 (0.715–0.892)	<0.001 ***	1.075 (0.968–1.193)	0.177
Medical insurance	No vs. Yes	0.693 (0.591–0.813)	<0.001 ***	0.932 (0.806–1.078)	0.343

Note: OR, odds ratio; CI, confidence interval. Estimates were pooled across 20 FCS multiple-imputation datasets using Rubin’s rules. Model 2, the primary confounder-adjusted model, included age, sex, marital status, residence, living arrangement, education, pre-retirement occupation, annual household income, pension insurance, and medical insurance. Pattern 3 was the outcome reference category and Class 5 was the intrinsic-capacity reference group. Other reference categories were female sex, unmarried status, rural residence, living alone, college or above education, farmers/others, annual household income ≥100,000 RMB, pension insurance = yes, and medical insurance = yes. * *p* < 0.05, ** *p* < 0.01, *** *p* < 0.001.

## Data Availability

The data that support the findings of this study are openly available from the Chinese Longitudinal Healthy Longevity Survey (CLHLS), conducted by the Center for Healthy Aging and Development Studies/National School of Development at Peking University. The data can be accessed through the Peking University Open Research Data Platform at https://opendata.pku.edu.cn/dataverse/CHADS (accessed on 17 October 2025). Researchers interested in using these data are required to register and submit a data use agreement for approval. The authors have obtained the necessary permissions to use the data for this study.

## Supplementary Materials

The following supporting information can be downloaded at: https://www.mdpi.com/article/10.3390/healthcare14203412/s1, Table S1: Comparison of characteristics between included and excluded participants; Table S2: Rubin-pooled multinomial logistic regression models and classification-confidence sensitivity analysis.
