# Supplementary material for: Associations Between Intrinsic Capacity Profiles and Unmet Home- and Community-Based Care Need Patterns Among Older Adults in China: A Cross-Sectional Study
Source: Healthcare (Basel). 2026 Oct 13;14(20):3412. doi: 10.3390/healthcare14203412 (PMC13704391; doi:10.3390/healthcare14203412)
Supplement: Supplementary file 1 [file healthcare-14-03412-s001.zip › healthcare-4488546-supplementary.pdf]

Supplementary Table S1. Comparison of characteristics between included and excluded participants

Characteristic	Category	Excluded (n = 5,469)	Included (n = 10,405)	Valid n (Excluded / Included)	Statistic	P value	Effect size
Age, years		92 (81, 100)	83 (75, 92)	5,461 / 10,405	U=19,261,385	<0.001	0.568
Sex	Male	2,019 (36.9)	4,906 (47.2)	5,469 / 10,405	χ^2=152.640	<0.001	0.098
	Female	3,450 (63.1)	5,499 (52.8)				
Marital status	Married	1,489 (28.2)	4,922 (47.7)	5,283 / 10,324	χ^2=548.835	<0.001	0.188
	Divorced/Widowed	3,733 (70.7)	5,323 (51.6)				
	Unmarried	61 (1.2)	79 (0.8)				
Residence	Urban	3,015 (55.1)	5,765 (55.4)	5,469 / 10,405	χ^2=0.111	0.739	0.003
	Rural	2,454 (44.9)	4,640 (44.6)				
Living arrangement	Not living alone	3,923 (85.9)	8,574 (82.4)	4,569 / 10,405	χ^2=27.508	<0.001	0.043
	Living alone	646 (14.1)	1,831 (17.6)				
Education	Illiterate	2,926 (63.0)	3,881 (43.8)	4,641 / 8,861	χ^2=461.906	<0.001	0.185
	Elementary school	1,123 (24.2)	3,092 (34.9)				
	Junior high school	276 (5.9)	982 (11.1)				
	Secondary school	186 (4.0)	564 (6.4)				
	College or above	130 (2.8)	342 (3.9)				
Occupation before retirement	Technical/management	373 (8.0)	1,053 (11.9)	4,653 / 8,827	χ^2=70.618	<0.001	0.072
	General employees	603 (13.0)	1,345 (15.2)				
	Farmers/others	3,677 (79.0)	6,429 (72.8)				
Annual household income	≤50,000 RMB	3,301 (70.4)	6,433 (66.6)	4,692 / 9,657	χ^2=23.677	<0.001	0.041
	50,001–99,999 RMB	534 (11.4)	1,334 (13.8)				
	≥100,000 RMB	857 (18.3)	1,890 (19.6)				
Pension insurance	No	2,165 (50.8)	5,236 (50.3)	4,259 / 10,405	χ^2=0.316	0.574	0.005
	Yes	2,094 (49.2)	5,169 (49.7)				
Medical insurance	No	632 (13.4)	1,141 (11.0)	4,721 / 10,405	χ^2=18.397	<0.001	0.035

	Yes	4,089 (86.6)	9,264 (89.0)				
Financial support from children, 1000 RMB		1.1 (0, 3.6)	1.6 (0, 4)	4,653 / 9,795	U=21,781,263	<0.001	0.036
Emotional support from children	No	831 (16.4)	1,483 (14.3)	5,067 / 10,389	χ^2=12.087	<0.001	0.028
	Yes	4,236 (83.6)	8,906 (85.7)				
Care support from children	No	620 (11.6)	1,015 (9.8)	5,332 / 10,404	χ^2=13.269	<0.001	0.029
	Yes	4,712 (88.4)	9,389 (90.2)				
BADL score		18 (12, 18)	18 (18, 18)	4,695 / 10,405	U=16,045,611.5	<0.001	0.771
Self-rated health		3 (3, 4)	3 (3, 4)	4,037 / 10,405	U=19,569,490	<0.001	0.134
Chronic disease	None	2,244 (41.0)	3,100 (29.8)	5,469 / 10,405	χ^2=204.191	<0.001	0.113
	Single	1,501 (27.4)	3,304 (31.8)				
	Two or more	1,724 (31.5)	4,001 (38.5)				

Note: Data are presented as n (%) for categorical variables and median (Q1, Q3) for continuous/ordinal variables. Percentages were calculated using available cases for each characteristic; therefore, valid denominators vary, particularly in the excluded group because missing key information contributed to sample exclusion. U denotes the Mann–Whitney U statistic; χ^2 denotes the Pearson chi-square statistic. Effect size is the absolute standardized mean difference (|SMD|) for continuous/ordinal variables and Cramér's V for categorical variables.

Supplementary Table S2. Rubin-pooled multinomial logistic regression models and classification-confidence sensitivity analysis

Panel A. Model 1: core sociodemographic and socioeconomic adjustment

Variable	Category / contrast	Pattern 1 vs Pattern 3 OR (95% CI)	P	Pattern 2 vs Pattern 3 OR (95% CI)	P
Intrinsic capacity profile	Class 1: Very Severe Multidomain Impairment	1.369 (1.001–1.874)	0.049*	1.315 (0.979–1.766)	0.069
	Class 2: Severe Multidomain Impairment	1.121 (0.904–1.391)	0.297	1.060 (0.865–1.300)	0.572
	Class 3: Moderate Multidomain Vulnerability	1.087 (0.931–1.268)	0.293	1.004 (0.866–1.163)	0.959
	Class 4: Cognition-Preserved Locomotion–Psychological Vulnerability	1.297 (1.129–1.489)	<0.001***	1.072 (0.937–1.228)	0.312
	Class 5: Comprehensive High-Functioning	Reference	—	Reference	—
Age	Per 1-year increase	0.995 (0.988–1.001)	0.089	0.996 (0.990–1.002)	0.149
Sex	Male vs Female	1.090 (0.977–1.217)	0.124	1.081 (0.972–1.202)	0.151
Marital status	Married vs Unmarried	0.984 (0.533–1.816)	0.958	0.699 (0.408–1.197)	0.192
	Divorced/Widowed vs Unmarried	0.877 (0.476–1.617)	0.674	0.714 (0.417–1.220)	0.218
Residence	Urban vs Rural	1.348 (1.212–1.499)	<0.001***	1.148 (1.039–1.269)	0.007**
Living arrangement	Not living alone vs Living alone	0.984 (0.852–1.136)	0.823	0.959 (0.839–1.096)	0.539
Education	Illiteracy vs College or above	0.787 (0.570–1.088)	0.147	0.956 (0.678–1.347)	0.796
	Elementary school vs College or above	0.696 (0.514–0.943)	0.020*	0.913 (0.656–1.271)	0.591
	Junior high vs College or above	0.681 (0.498–0.931)	0.016*	1.011 (0.723–1.414)	0.949
	Secondary school vs College or above	0.669 (0.483–0.926)	0.015*	1.075 (0.761–1.518)	0.681
Occupation before retirement	Technical/management vs Farmers/others	0.672 (0.541–0.834)	<0.001***	0.590 (0.478–0.729)	<0.001***
	General employees vs Farmers/others	0.837 (0.715–0.980)	0.027*	0.619 (0.525–0.728)	<0.001***
Household annual income	≤50,000 RMB vs ≥100,000 RMB	0.912 (0.795–1.046)	0.186	1.407 (1.224–1.618)	<0.001***
	50,001–99,999 RMB vs ≥100,000 RMB	1.269 (1.072–1.502)	0.006**	1.082 (0.904–1.296)	0.390

Panel B. Model 3: extended adjustment model

Variable	Category / contrast	Pattern 1 vs Pattern 3 OR (95% CI)	P	Pattern 2 vs Pattern 3 OR (95% CI)	P
Intrinsic capacity profile	Class 1: Very Severe Multidomain Impairment	1.273 (0.912–1.777)	0.156	1.239 (0.906–1.695)	0.180
	Class 2: Severe Multidomain Impairment	1.066 (0.852–1.334)	0.578	1.000 (0.809–1.235)	0.997
	Class 3: Moderate Multidomain Vulnerability	1.030 (0.878–1.208)	0.716	0.958 (0.824–1.115)	0.580
	Class 4: Cognition-Preserved Locomotion–Psychological Vulnerability	1.208 (1.047–1.393)	0.010**	1.017 (0.885–1.169)	0.807
	Class 5: Comprehensive High-Functioning	Reference	—	Reference	—
Age	Per 1-year increase	0.996 (0.990–1.003)	0.244	0.998 (0.992–1.004)	0.438
Sex	Male vs Female	1.111 (0.994–1.241)	0.063	1.057 (0.949–1.176)	0.314
Marital status	Married vs Unmarried	0.806 (0.429–1.517)	0.505	0.814 (0.468–1.415)	0.465
	Divorced/Widowed vs Unmarried	0.711 (0.376–1.343)	0.293	0.853 (0.488–1.491)	0.577
Residence	Urban vs Rural	1.327 (1.192–1.478)	<0.001***	1.167 (1.055–1.291)	0.003**
Living arrangement	Not living alone vs Living alone	0.986 (0.853–1.140)	0.854	0.982 (0.858–1.124)	0.792
Education	Illiteracy vs College or above	0.834 (0.602–1.156)	0.276	0.932 (0.660–1.318)	0.692
	Elementary school vs College or above	0.729 (0.536–0.991)	0.044*	0.906 (0.649–1.264)	0.559
	Junior high vs College or above	0.709 (0.517–0.973)	0.033*	1.013 (0.722–1.421)	0.941
	Secondary school vs College or above	0.684 (0.492–0.950)	0.024*	1.078 (0.763–1.523)	0.671
Occupation before retirement	Technical/management vs Farmers/others	0.632 (0.506–0.789)	<0.001***	0.624 (0.502–0.776)	<0.001***
	General employees vs Farmers/others	0.786 (0.665–0.929)	0.005**	0.660 (0.556–0.783)	<0.001***
Household annual income	≤50,000 RMB vs ≥100,000 RMB	0.931 (0.810–1.070)	0.311	1.324 (1.151–1.523)	<0.001***
	50,001–99,999 RMB vs ≥100,000 RMB	1.272 (1.074–1.508)	0.005**	1.060 (0.885–1.268)	0.527
Pension insurance	No vs Yes	0.803 (0.719–0.897)	<0.001***	1.059 (0.953–1.176)	0.287
Medical insurance	No vs Yes	0.707 (0.602–0.830)	<0.001***	0.933 (0.806–1.080)	0.356
Financial support from children	Per 1000 RMB increase	1.011 (1.003–1.018)	0.006**	0.985 (0.975–0.995)	0.004**
Emotional support from children	No vs Yes	1.030 (0.851–1.246)	0.765	1.337 (1.123–1.592)	0.001**

Care support from children	No vs Yes	0.738 (0.588–0.925)	0.009**	0.854 (0.694–1.051)	0.137
Chronic disease	None vs ≥2 conditions	0.936 (0.825–1.062)	0.302	1.281 (1.137–1.445)	<0.001***
	Single vs ≥2 conditions	0.960 (0.853–1.081)	0.504	1.144 (1.019–1.283)	0.022*
BADL score	Per 1-point increase	0.996 (0.963–1.030)	0.809	1.025 (0.991–1.060)	0.150
Self-rated health	Per 1-point increase	0.885 (0.835–0.937)	<0.001***	0.845 (0.799–0.893)	<0.001***

Panel C. High-classification-confidence sensitivity analysis

Variable	Category / contrast	Pattern 1 vs Pattern 3 OR (95% CI)	P	Pattern 2 vs Pattern 3 OR (95% CI)	P
Intrinsic capacity profile	Class 1: Very Severe Multidomain Impairment	1.803 (1.242–2.618)	0.002**	1.317 (0.940–1.845)	0.109
	Class 2: Severe Multidomain Impairment	1.252 (0.949–1.652)	0.112	1.056 (0.829–1.345)	0.660
	Class 3: Moderate Multidomain Vulnerability	1.182 (0.959–1.456)	0.118	1.016 (0.845–1.221)	0.867
	Class 4: Cognition-Preserved Locomotion–Psychological Vulnerability	1.444 (1.157–1.803)	0.001**	1.134 (0.927–1.387)	0.220
	Class 5: Comprehensive High-Functioning	Reference	—	Reference	—
Age	Per 1-year increase	0.997 (0.989–1.006)	0.548	0.998 (0.991–1.005)	0.604
Sex	Male vs Female	1.052 (0.912–1.214)	0.488	1.210 (1.065–1.374)	0.003**
Marital status	Married vs Unmarried	1.357 (0.564–3.264)	0.496	0.678 (0.354–1.300)	0.242
	Divorced/Widowed vs Unmarried	1.050 (0.437–2.523)	0.914	0.662 (0.346–1.266)	0.213
Residence	Urban vs Rural	1.397 (1.214–1.608)	<0.001***	1.190 (1.054–1.344)	0.005**
Living arrangement	Not living alone vs Living alone	0.914 (0.755–1.105)	0.353	0.951 (0.807–1.120)	0.547
Education	Illiteracy vs College or above	0.816 (0.542–1.229)	0.329	1.137 (0.755–1.710)	0.539
	Elementary school vs College or above	0.704 (0.483–1.025)	0.067	1.054 (0.713–1.557)	0.793
	Junior high vs College or above	0.669 (0.456–0.982)	0.040*	1.111 (0.749–1.649)	0.600
	Secondary school vs College or above	0.716 (0.485–1.058)	0.094	1.213 (0.811–1.817)	0.347
Occupation before retirement	Technical/management vs Farmers/others	0.694 (0.521–0.925)	0.013*	0.640 (0.495–0.828)	<0.001***
	General employees vs Farmers/others	0.794 (0.639–0.986)	0.036*	0.614 (0.499–0.755)	<0.001***
Household annual income	≤50,000 RMB vs ≥100,000 RMB	0.997 (0.830–1.198)	0.978	1.439 (1.216–1.702)	<0.001***
	50,001–99,999 RMB vs ≥100,000 RMB	1.350 (1.089–1.675)	0.006**	1.094 (0.885–1.353)	0.407
Pension insurance	No vs Yes	0.736 (0.637–0.851)	<0.001***	1.067 (0.940–1.211)	0.314
Medical insurance	No vs Yes	0.649 (0.525–0.801)	<0.001***	0.928 (0.779–1.106)	0.404

Sensitivity analysis uses Model 2 covariates and is restricted to participants with maximum posterior probability ≥0.80 for both the intrinsic-capacity profile and unmet-need pattern classifications (n = 6,706).

Note: Values are ORs (95% CIs) pooled across 20 FCS multiple-imputation datasets using Rubin's rules. Pattern 3 is the outcome reference category. Class 5 is the intrinsic-capacity reference profile. Other reference categories are female sex, unmarried status, rural residence, living alone, college or above education, farmers/others, annual household income ≥100,000 RMB, pension insurance = yes, and medical insurance = yes where applicable. * $p < 0.05$, ** $p < 0.01$, *** $p < 0.001$.
